# Managing constraint: frugal opposition to European fiscal solidarity

**DOI:** 10.1080/13501763.2024.2332697

**Published:** 2024-03-24

**Authors:** David Bokhorst, Magnus G. Schoeller

**Affiliations:** a European University Institute, San Domenico di Fiesole, Italy; b Centre for European Integration Research (EIF), Department of Political Science, University of Vienna, Vienna, Austria

**Keywords:** Politicisation, European integration, Governments, Next Generation EU

## Abstract

The management of the COVID-19 crisis and, in particular, the Next Generation EU fund have shown that European leaders can find integrationist policy solutions despite increasing politicisation at home where democratic constraints may lead to a feared ‘multilevel politics trap’. Therefore, we ask whether and how national governments can manage such constraints and thus spring or avoid the trap. Theoretically, we argue that the agency of governments is a crucial factor for understanding the varying dynamics of politicisation in regional integration, as governments can raise or lower domestic audience costs by strategically interacting with their parliament or media. Empirically, we probe the plausibility of our theoretical propositions by examining constraint management and position-taking in Austria and the Netherlands in the context of European fiscal solidarity. Our results show that there is no inevitably self-reinforcing multilevel politics trap but that the effects of domestic constraint are, to a considerable extent, contingent on the agency of national governments.

## Introduction

As European integration advances further and increasingly enters into areas of core state powers, so too does the politicisation of European policymaking in domestic arenas. Today, European Union (EU) politics is widely debated and contested (Börzel & Risse, 2018). In times of an EU polycrisis, European leaders face a higher demand for European solutions to common problems and more politicised debates at home. In reflecting on this predicament, Zeitlin et al. (2019) argued that politicisation may limit the EU’s capacity to deliver effective solutions (see also Hutter & Kriesi, 2019). This, in turn, could lead to ‘a multilevel politics trap’ (Nicoli & Zeitlin, this issue), a potential scenario where the EU, in times of need, is paralysed along political divides, thus sapping output legitimacy and fueling a vicious cycle of even more scepticism over the inability of the EU to act. In the years before the COVID-19 crisis, political divides indeed seemed to stifle decision-making. On fiscal integration, in 2018, eight northern Member States, known as the New Hanseatic League, formed a bloc to challenge the more solidaristic vision of Economic and Monetary Union (EMU) governance emerging from a Franco-German compromise (Schoeller, 2021; 2022). With the refugee and migration crisis following similar patterns, those studying the polycrisis asked whether a politics trap might tighten over time with entrenched positions along stable divides (Zeitlin et al., 2019). Pathways out of potential paralysis were sought by some in more structural solutions for the Union, for example, by encouraging differentiated forms of integration to keep the EU governable (de Vries, 2018).

The COVID-19 crisis and ensuing policy response challenged the assumptions of the multilevel politics trap at their core. The significant fiscal expansion of the 750-billion-euro Next Generation EU (NGEU) is testimony to European leaders’ ability to overcome the rifts of politicisation at home and choose common rather than differentiated solutions in times of need. From the outset, the COVID-19 response alluded more to a classic neofunctionalist understanding of a Europe forged through crisis. It suggests that we should conceive of the trap as closed only under normal times but with the ability to open up if crisis pressure is strong enough to make all entrenched positions malleable. While it is undeniable that the nature of the crisis played a significant role in explaining the compromise on NGEU, the neo-functionalist reading is still unsatisfactory. It leaves out the important role of agency in understanding crisis dynamics in more granular terms and leaves little space for democratic deliberation and legitimate contestation. While all Member States and parliaments accepted the NGEU compromise, this was certainly not without controversy and contestation. Austria, Denmark, Sweden and the Netherlands, known as the Frugal Four, maintained fundamental opposition to the very end. In this paper, we seek to bring strategic agency under politicisation pressure into the equation.

Events around NGEU stress the need to re-evaluate the theoretical underpinnings of the concept of a multilevel politics trap as a potential pitfall of increased politicisation in the EU. A stifling trap was avoided despite continuous fundamental opposition, and significant integration followed. This calls into question the level and nature of domestic constraint due to politicisation and strategic agency of governments in response to this pressure. As such, in this paper, we ask: to what extent and how does domestic politicisation lead to constraints on governments’ ability to seek compromise? And, how do governments manage this constraint in times of crisis? To examine these questions, we look at two core sceptics in the debate on fiscal solidarity, the Netherlands and Austria, and analyse the domestic debate and ensuing two-level game in the government’s response to understand how constraint was managed.

In this paper, we theorise how governments can either ‘spring the trap’ or avoid it by interacting with their national parliament and/or the media. Empirically, we draw on in-depth interviews and official documents to investigate how the Dutch and Austrian governments shape their EU policy in the face of domestic discontent.

## Theory: understanding the role of strategic agency under politicisation pressure

The ever-advancing nature of integration despite democratic opposition has long puzzled scholars. While classical accounts of neofunctionalism held high hopes of politicisation as a driving force for EU integration (Schmitter, 1969), the rise of Eurosceptic parties since the early 2000s made way for a more sober reading. The ensuing fragmentation in EU politics, with European elites more wary of sceptic publics at home, has famously been termed by postfunctionalists Hooghe and Marks as politics under a ‘constraining dissensus’ (2009). In our view, the steady advance of integration, most notably with initiatives such as NGEU, throws into stark relief portrayals of an EU governed by a fundamental dissensus. However, we agree with Hooghe and Marks that politicisation may lead to constraints on the ability to compromise. Politicisation is generally understood as a function of the growing domestic salience of EU politics, a polarisation of opinions and positions and the expansion of actors and audiences engaged with EU affairs (de Wilde et al., 2016). On more constitutive issues about the widening and deepening of EU integration, politicisation is a growing phenomenon (Grande & Hutter, 2016), leading to an intensification of domestic political divides between proponents of integration and demarcation (Hutter & Kriesi, 2019). Politicisation thus provides fertile ground for mobilisation against integrationist initiatives. Governments act responsively in their Council positions when issues are salient domestically (Hobolt & Wratil, 2020) and tend to take a firmer stance when elections are looming (Kleine & Minaudier, 2019). The classical insights on ‘two-level game’ theory of Putnam (1988) point out that governments can also capitalise on domestic constraint by using it as a credible threat in international negotiations to strengthen their position.

These studies suggest that governments anticipate potential opportunity or reputational loss and act strategically. In responding to bottom-up pressure arising from politicisation, EU-level actors can choose to capitalise on domestic pressure to advance their substantive goals or to depoliticise through framing or package deals to avoid visible conflict and contestation (Schimmelfennig, 2020). As such, in their 2020 JEPP Special Issue on ‘EU actors under pressure’, Bressanelli et al. (2020) conclude that there is considerable space for strategic agency in EU politics, which, they consider, challenges the constraining part of the constraining dissensus thesis. Zeitlin et al., too, did not conceive of their multi-level politics trap thesis as deterministic without a role for strategic agency but focused on different politicisation dynamics in the face of multiple interwoven crises and possible escape pathways. We may consider that a depoliticisation strategy, as Schimmelfennig theorised, is less feasible in deep crises where salience is high. Also, Truchlewski and Schelkle (2022), for example, showed that, in the early phase of the COVID-19 crisis, leaders opted for politicisation strategies in terms of expanding audiences and raising salience by posting op-eds in newspapers in other Member States to argue for a wide range of reform options, with the Commission only coming in with a proposal at a later stage. In short, the expanding European political space implies that governments must respond strategically to multiple reform options simultaneously, thus complicating the ensuing two-level game they have to play in both the European and domestic political arenas.

In this paper, we build on the insights of this literature but also conclude that both the level and nature of constraint and strategic government agency in facing complex crises require further examination. In addressing these issues, we take as a starting point that government actors’ paramount motive is to increase their popularity at home and avoid reputational costs. Building on both the multilevel politics trap concept and on Schimmelfennig’s politicisation/depoliticisation thesis, we conjecture that, faced with democratic discontent at home *and* pressure to compromise at the European level, government actors can either ‘spring the trap’ or seek to ‘avoid or circumvent the trap’. This dichotomy assumes that there is entrapment pressure arising from politicisation. With entrapment, we mean the process of raising ex-ante costs for European integrationist initiatives. Democratic discontent pressures policymakers to become more concrete in voicing ex-ante opposition to further integration steps, which is the constraining effect of politicisation that is central to our study. The more concrete and fundamentally the opposition is expressed – by drawing red lines and threatening with vetoes – the deeper the ensuing trap and the harder it will be to compromise or to sell the compromise at home ex-post.

Springing the trap means that policymakers portray themselves as powerful defenders of their state’s interests and raise domestic audience costs of compromise to strengthen their position in the EU negotiations and remain popular at home. Springing the trap would indeed set in motion the proclaimed multilevel politics trap dynamic at the European level. Sometimes, however, it may provide a larger electoral advantage if governments avoid or circumvent the trap rather than springing it, as the anticipated costs of a negotiating loss would lead to reputational damage. This second way of managing constraint is relevant if it becomes clear that specific goals cannot be reached at the European level or if the negative externalities of preventing an agreement are considered too high. In such a situation, governments may seek to limit the political costs in the domestic debate by avoiding statements of fundamental opposition on which they are unlikely to be able to deliver. In this case, the response to entrapment pressure would be to maintain an oppositional position in the debate but avoid or circumvent red lines and leave space – or constructive ambiguity – for an eventual compromise. Avoiding the trap means that governments refrain from statements of fundamental opposition, while circumvention means they seek to manoeuvre around pre-existing red lines.

We expect that the choice to spring or avoid/circumvent the trap depends on where governments see the greater electoral advantage.^1^ This is where coalitions in the Council come in, as we expect governments to be more likely to spring the trap if they anticipate sufficient support from others to deliver on their promises. In contrast, isolated positions may achieve the opposite. Furthermore, in a situation of contestation and salience, powerful parliaments are unlikely to let avoidance occur, and governments may face a more difficult task of circumvention.

We add one further layer to the analysis: the agency of coalition parties in parliament. Their role is usually not accounted for when examining two-level game dynamics, but they are key actors in establishing constraints. Coalition parties hold the majority in parliament and are, therefore, the guardians on whether motions or other forms of constraint are introduced. We may assume that they act responsively to electoral incentives, though, unlike opposition parties, coalition parties face the same predicament in their actions as the government itself. If they constrain too much ex-ante, they may partly be responsible for the reputational damage to their government, which they will seek to avoid. Here, it should be noted that, rather than passive receivers of instructions, governments also actively engage with parliaments by sharing information to set realistic expectations of the outcome. In other words, we expect that constraint is not an exogenous force but a process that governments actively manage.

Hence, we assume that governments can manage constraints arising from politicisation by manipulating domestic audience costs (see Slantchev, 2006). More precisely, they can raise audience costs if they want to bind their hands and thus increase their bargaining power in EU negotiations. This is the case if they see a greater electoral advantage in portraying themselves as defenders of the national interest. Alternatively, they can lower audience costs if they want to increase their room for manoeuvre at the European level and thus allow for compromises to solve common problems. This is the case if they expect that the functional disadvantages of non-agreement at the EU level outweigh the possible electoral advantages at home. In both cases, governments have two channels to manipulate audience costs: parliaments and media. In parliament, the coalition parties can either try to bind their government (e.g., through motions) and strictly scrutinise its actions (= raising audience costs), or they can unconditionally back their government, prevent binding motions and abstain from public scrutiny (= lowering audience costs). Likewise, the government can strategically place specific issues in the media to raise audience costs, or it can prevent certain information from being passed to the media or try to influence coverage to keep audience costs low.

## Methodology and case selection

To probe the plausibility of our theoretical argument and assess how governments manage constraint, we look at frugal states in the debate on EU fiscal solidarity.^2^ This is because the dynamics of a multilevel politics trap are arguably most likely to play out in redistributive conflicts about transnational solidarity. Politicisation, in terms of both salience and contestation, should be highest in those states that would be the relative losers in such solidarity solutions, hence the ‘net payers’ or ‘creditors’. Indeed, frugal states are most opposed to deeper integration in fiscal policy, as they favour national responsibility over European transfers.

From the EMU Choices database (Wasserfallen et al., 2019) and the follow-up on NGEU positions by Truchlewski and Schelkle (2022), we can identify Austria and the Netherlands among those countries with the most frugal preferences. Unlike other frugal nations, such as Denmark and Finland, they are both eurozone members *and* part of the Frugal Four coalition, which vocally opposed fiscal solidarity in COVID-19 crisis management. Moreover, both countries have a similarly high vote share of hard Eurosceptic parties in parliament (de Vries, 2018), so we may expect more vocal opposition at home. In the comparative literature on the politicisation of EU issues, the Netherlands and Austria rank slightly above the average (Hoeglinger, 2016; Hutter & Kriesi, 2019). In terms of formal scrutiny rights of the national parliaments, both countries rank roughly the same, with Austria having more substantial formal rights of constraint and the Netherlands described as more fully involved in scrutiny (Auel, 2015). From the outset, we may, therefore, consider Austria and the Netherlands as ‘most likely’ cases (Levy, 2008, pp. 12–13) in having several relevant and roughly similar features to test whether and how a context of politicisation may lead to constraint and how constraint is managed. The case comparison serves to find additional patterns for inductive theorisation, while in the conclusions, we discuss to what extent our findings translate to other contexts.

Hence, this paper is based on a structured case comparison studying strategic two-way interaction at two levels. The first level concerns interactions between governments and domestic arenas of contestation: parliament and the media. The second level is the interaction between governments and the EU institutions. To be clear, we do not seek to explain how the NGEU deal was passed, which has already been described in detail (Smeets & Bekius, 2022; Truchlewski et al., 2021), but to analyse how domestic constraints influenced position-taking by Austria and the Netherlands and how a strong or weaker standing in the Council influenced how governments managed constraints at home. The emphasis is on domestic two-way interaction as the understudied phenomenon.

Since we already know that NGEU got passed, and thus, constraint was ultimately managed, we take a wider perspective in terms of the timeline of examination. We analyse strategic interaction from the pre-NGEU debate on fiscal solidarity, starting with the debate on the Eurozone budget (later called the Budgetary Instrument for Convergence and Competitiveness – BICC) and the rise of the Hanseatic League, to the COVID-19 crisis when crisis pressure rose, and the frugals morphed into the Frugal Four minority position. We include the immediate post-NGEU aftermath to check if we find noticeable changes in strategic behaviour.

In terms of data collection, we assess the strategic choice of actors by analysing the relevant parliamentary debates and government documents during the Hanseatic League period and around the NGEU deal. In particular, we look at parliamentary motions and statements of (frugal) coalition parties in setting up ex-ante barriers to compromise and how these changed during the NGEU negotiation and in its aftermath. In addition, we examine strategic choice by looking at statements of relevant leaders in the media or speeches that may imply an increase in reputational costs in case of an EU compromise. We have created an online appendix with more details and links to the government documents we used. We triangulate these sources with 20 semi-structured elite interviews conducted with politicians, government officials, and media representatives who deal directly with EU policy-making at the national and/or European level, including those directly involved with the NGEU negotiations and parliamentary staff, to help us achieve accuracy in the document analysis. Among other issues, we asked interviewees to explain how parliamentary action or media coverage has affected the government’s strategic choices, or *vice versa*, and why certain strategic decisionsin terms of springing or avoiding/circumventing the trap were made. In conducting interviews, we sought to ensure internal validity by guaranteeing interviewees’ anonymity and avoiding questions implying social desirability or causal relationships.

## The Netherlands

### Constraint and its management pre-NGEU

The Netherlands scores well above the average regarding public support for EU membership (Eurobarometer, 2022, p. 80). But at the level of specific policies, contestation, polarisation, media attention and opposition can take on very pronounced and loud forms. Nowhere is this more true than on the issue of fiscal solidarity. In Parliament, most MPs favour the strict application of budgetary rules to prevent the EU from moving in the direction of fiscal transfers and try to intervene as early in the process as possible. It is emblematic of the type and intensity of politicisation that parliamentary debate is not just confined to debating opposing views on European initiatives but also extends to policy in other Member States. For example, in the 2018 budgetary discussion between the Commission and Italy, the Dutch parliament – especially the centre-right coalition parties – actively steered the government’s position, calling upon the Minister of Finance to raise the issue with colleagues (TK, 2018; 2018a), for a Hanseatic League statement on Italy (TK, 2019), for Prime Minister Rutte to get involved (TK, 2019c) and even targeting the European Commission directly to question its models and decisions (TK, 2019; 2019a; 2019b; 2019d; 2019e). In one parliamentary debate no less than 181 references to ‘Italy’ appear (TK, 2018b). As an indication of the constant salience of European fiscal affairs, 91 newspaper articles directly referencing the Italian budget saga can be found in the five biggest newspapers.^3^

As the example illustrates, domestic scrutiny and debate about European fiscal policies are high in parliament and receive constant media attention. Sceptical parties anticipate very early in the process that European developments go in an undesired direction and use political means to push the government into voicing opposition. With the permissive consensus on EU affairs waning in the early 2000s, the Dutch parliament has seen a continuous process of evaluation of its own procedures to tighten ex-ante control on governments’ position in EU affairs (TK, 2002; 2011; 2014). This has led to many EU-related debates and extensive information provision before and after each Council meeting and EU proposal. For example, in 2019, the budgetary instrument BICC was discussed in eight three-hour debates plus two plenary debates,^4^ and the first outline of the instrument was followed by 144 written parliamentary questions (TK, 2019g). Parliament also uses motions to tighten ex-ante control. Motions are statements of majority support in parliament and, while not directly legally binding, they are not easy to ignore politically and de facto constrain the government by making red lines more explicit and precise. The two most important red lines on European fiscal solidarity are the rejection of Eurobonds and of automatic stabilisation (TK, 2012; 2017; 2018d). In 2014, a wide parliamentary majority adopted a motion calling upon the government to ‘forcefully reject *any type* of European debt obligations’ (TK, 2014a). On the BICC, Parliament passed a motion that if the BICC did not abide by Parliament’s wishes the Netherlands would have to opt out (TK, 2019f). Also here, media salience around the issue was present, with 94 newspaper articles referencing it.^5^

Whether intense parliamentary scrutiny and motions to guard red lines also effectively constrain the government is a nuanced matter. The government itself has long agreed with the parliamentary majority that stabilisation and joint borrowing are out of the question (TK, 2013). Behind the scenes, coalition MPs negotiate with ministers to ensure that their motions do not move too far from the government’s position. But in terms of constraint, motions are not entirely futile either, as they codify – often in detail – the position early in the process and act as an accountability device increasing the political cost of compromises. Interviewees describe motions also as a beneficial constraint, which ensures transparency and consistency of the position and which may help in EU negotiations (Interviews NL1, NL2, NL3). It is in light of very tight parliamentary scrutiny that we can understand Finance Minister Hoekstra’s tough negotiation tactics around the BICC. As a sign of ‘springing the trap’, Hoekstra presented himself as a leader of the Hanseatic League, who would not shy away from holding Eurogroup colleagues hostage until deep in the night to protect Dutch red lines on stabilisation (Interviews NL1, NL2, NL3; Verdun, 2021). With the support of the Hanseatic League, Hoekstra was able to build up national popularity and international fame as ‘Mr. No’ (Interview NL6; Politico, 2020). The negotiation outcome on the BICC, where French hopes of a big new budget morphed into a 25 billion reform conditionality instrument within the existing EU budget, was celebrated by Dutch officials as a victory on all fronts (Schoeller, 2021).

In sum, in the pre-NGEU period, the government faced assertive parliamentary scrutiny and motions on red lines in the context of media salience. In this highly politicised context, government actors also took a leading role in opposing integrationist initiatives. They presented themselves as skilful protectors of national red lines, thus clearly springing a multilevel politics trap.

### Constraint and its management in a crisis context

At the start of the COVID-19 crisis, the Dutch position on fiscal solidarity appeared entrenched and codified in motions and government statements. However, early in the pandemic, the Dutch realised it was impossible to maintain a hardliner position when Hoekstra’s hawkish statements led to a severe outcry among Southern European leaders. They made it clear that with hospitals overflowing, now was not the time for classic moral hazard rhetoric (Smeets & Bekius, 2022). Their response fuelled domestic debate and led to reputational damage. Politicians understood that solidarity was called for but also wanted to protect their red lines. The solution was to try to control the framing of the crisis as a health emergency, but not an economic crisis, which needed support, but not structural economic intervention (Interview NL1). As such, civil servants were instructed to ensure that the proposal for fiscal solidarity to mitigate unemployment risks (SURE) would also focus on support for health. Hoekstra feared that an unemployment-only instrument would be seen in Parliament as the prequel to a stabilisation instrument, thus going against previously set red lines (Interview NL1).

While centre-right MPs in Parliament had spent most of 2018 and 2019 positioning themselves almost every month vocally against ‘the inevitable transfer union’, during the early phases of the COVID-19 crisis – when ideas on Coronabonds, perpetual bonds and other far-reaching proposals were flowing in the European political space – the constraint from Parliament was surprisingly low. In April 2020, a motion was passed that called for solidarity based on immediate extra support while maintaining that this support cannot imply debt mutualisation based on Eurobonds (TK, 2020b). In the following crucial weeks, no debates were organised due to lockdowns until just before the European Council meeting in July 2020, when the NGEU deal was already more or less assured. In this final debate, centre-right MPs took a conciliatory tone, focusing on substantive issues such as conditionality requirements and motions from populist radical right parties calling for vetoes were rejected (TK, 2020). A wide majority in Parliament eventually passed the Own Resources Decision underlying the NGEU deal (TK, 2020a).

Meanwhile, at the European level, Dutch negotiators focused all their efforts on broadening the mandate of SURE and protecting the European Stability Mechanism as an EMU-only instrument. Still, they were caught by surprise when France and Germany pushed for a new fund. As soon as the Eurogroup statement of 9 April 2020 included the crucial phrase that the EU would be looking for ‘innovative financial instruments’ it became apparent to Dutch civil servants that a substantial fund was inevitable (Interviews NL1, NL2, NL3). The Hanseatic League had never been a coalition with fundamentally unified views, so no real effort was made to bring it on line. Negotiators realised immediately that they were in a minority position, with only the Frugal Four left, and from there on, the strategy shifted from fundamental opposition to damage control and pragmatism, focused on hardening conditionality requirements and trying to achieve a higher proportion of loans versus grants (Interviews NL1, NL2, NL3).

Whereas the Netherlands positioned itself as an absolute hardliner against any type of European debt, this position became surprisingly malleable in a crisis context. Actors on the side of both the government and the centre-right sceptics in parliament anticipated reduced room for manoeuvre and chose to attune their opposition to lower reputational damage. As admitted by one interlocutor, ultimately, what matters in the debate is that PM Rutte, before a Council meeting, indicates where the compromise is likely to land (Interview NL1). The Coronabonds and Eurobonds discussion was a useful decoy in *circumventing a trap* whilst sticking to pre-agreed red lines. After the NGEU deal, Rutte could argue that the bonds underlying the NGEU were *strictu sensu* not Eurobonds, as they did not contain so-called *joint and several liability,* so no red line was crossed. The one-off character furthermore assures it is not a ‘debt union’ (TK, 2020a), a concept that parliament had never really defined. Meanwhile, the conditionality model of the RRF was based on what the Dutch had propagated since 2013 (Bokhorst & Corti, 2023), and Rutte had assured an extra Council check on payments to give parliament a sense of control in execution.

Interestingly, in the immediate aftermath of the NGEU deal, Parliament’s coping strategy was to double down on its pre-NGEU strategy to prevent the situation from happening again. In February 2022, Parliament adopted a motion to fortify its red line on joint borrowing, this time not focusing on Eurobonds, but on common debt issuance for new European projects in general (TK, 2022). This was followed with a motion arguing that the Recovery and Resilience Facility cannot be made permanent and that any type of structural financing mechanism is unacceptable (TK, 2022a) and a motion arguing that the Netherlands would have to opt out of any proposals for new common debt-based funds with a structural character (TK, 2023). Again, this looks like springing the trap, but governmental agency is a crucial factor. A new Minister from the Europhile D66 party entered the Finance Ministry in 2021. Contrary to her predecessor she avoided any reference to the Hanseatic League or Frugal Four, but rather chose to be a bridge builder with countries in Southern Europe (Rijksoverheid, 2022; NRC, 2023). Even a Europhile Minister cannot easily move beyond the constraints of Parliament on joint borrowing, even if the tone in government documents shifted from red lines to principles (TK, 2022b). However, the motions cannot be read as a full trap either, as the word ‘structural’ – emphasised in all motions – leaves open the possibility for one-off funds in the future.

In sum, while Dutch policymakers govern under a certain degree of constraint, ultimately, in deep crises, the reputational costs of vetoing a deal are deemed as too high, which signals that there is no fundamental dissensus. Rather, policymakers skilfully manoeuvred to circumvent a trap, with the Eurobonds as an important decoy in maintaining credibility in the face of red lines.

## Austria

### Constraint and its management pre-NGEU

Austria is a more Eurosceptic country than the Netherlands (Eurobarometer, 2022, p. 80). Indeed, since the country’s EU accession, Austrian approval rates for membership have been below the EU average (Meyer, 2023). However, while there is high polarisation regarding the desirability of European integration as such, many concrete EU issues remain ‘under the radar’ of the Austrian public and are difficult to place in the media (Interviews AT2, AT4, AT7, AT9, AT11, AT12; cf. Hurrelmann et al., 2015). There are salient EU issues that lead to contestation, most notably migration (Interviews AT2, AT9). On EMU and fiscal governance, however, our interviewees widely perceive of politicisation as low (Interviews AT1, AT2, AT3, AT4 AT6, AT7, AT8).^6^

Hence, while fiscal solidarity is contested and largely framed negatively (Auel & Schmidt, 2022), concrete EU budget and EMU matters receive little public attention (Interviews AT1, AT4, AT8, AT9). Moreover, media coverage of concrete EU issues is reported as relatively small by both government officials and journalists involved (Interviews AT4, AT7, AT8, AT11, AT12). Here it should be noted that the Austrian newspaper scene is dominated by tabloids, particularly the *Kronen-Zeitung,* whose circulation in 2021 exceeded that of the largest nationwide non-tabloid newspaper (*Kurier*) by a factor of 5.5*.*^7^ Newspapers that provide high quality journalism on EU issues, such as *Der Standard* or *Die Presse*, remain far behind the tabloids in terms of reach (Interviews AT2, AT6, AT7, AT9).

The low media salience of specific EU fiscal governance issues corresponds to the relatively small influence of the Austrian Parliament on these issues. Formally, the Austrian parliament has even more rights to bind the government than the Dutch parliament (Auel, 2015). In addition to subsidiarity control and the political dialogue with EU institutions, parliament can issue opinions (*Stellungnahme*) asking a minister or the Chancellor to take a certain position in the (European) Council. Such an opinion can even be binding if it refers to an EU proposal that entails the adoption of a national legal act. *De jure*, the Austrian Parliament can, therefore, intervene ex-ante in EU decision-making.^8^ *De facto*, however, it is highly unusual that an opinion would differ from the government’s preferences since government parties hold the majority in the competent committee (except for technocratic or minority governments). If a motion for a binding opinion is adopted nevertheless, it is virtually never without the prior approval of the responsible minister (Interview AT3). In practice, the instrument of parliamentary opinions, therefore, has hardly any constraining effect on the government’s EU policy. Specifically, on NGEU and the Frugal Four, we found no passed parliamentary motions, let alone inter-institutional agreements that would bind or even entrap the Austrian government. Beyond direct constraint, interviewees are in broad agreement that the Austrian Parliament has no significant influence on the government’s EU policy nor any effective ex-post scrutiny (Interviews AT1, AT2, AT3, AT5, AT6, AT7, AT8; see e.g., Auel et al., 2016; Miklin, 2015, p. 3). Consequently, parliament plays a rather reactive role. For example, when the Austrian government decided not to join the New Hanseatic League, even though it would have been a suitable format for representing Austrian preferences (see Schoeller, 2022; Schoeller & Falkner, 2022), this was not even discussed in Parliament.

Turning to the side of government, we would expect the Austrian government to be a vocal participant in the European debate, given the prominent role of Euroscepticism and domestic opposition to fiscal solidarity. However, due to the comparatively weak role of the media and parliament regarding EU issues, the Austrian government is rather unconstrained. Unlike the Netherlands, we find no official documents expressing fundamental opposition or even red lines. On the contrary, the EU chapter of the government programme has a very constructive tone. With the possible exception of rejecting the free trade agreement with Mercosur, no red lines can be identified (Bundeskanzleramt 2020, pp. 124–128). While generic Eurosceptic statements are common in Austrian politics, government members expressing fundamental opposition to proposed EU policies are rare (Interviews AT10, AT12). When such statements are made, they usually refer to areas where supposed Austrian interests should be protected, such as EU financing questions, migration, and competence transfers to Brussels. Usually, they are not a reaction to demands raised by the Parliament or the media, but proactive statements by politicians themselves, arguably to exploit the mobilisation potential of Eurosceptic voters (see Auel & Schmidt, 2022; Meyer, 2023; Interviews AT2, AT3, AT6, AT8, AT9, AT10, AT11, AT12, AT13). For example, when in February 2020, Chancellor Kurz threatened to veto the Commission’s proposal for the Multiannual Financial Framework (EU budget) if the Commission did not revise downward the proposed contributions, he was not pressed by the public, media, or Parliament to take such a firm position.^9^

Hence, in questions of EU fiscal governance, the government’s strategic behaviour in voicing opposition is rather reactive and passive. Austria relies on the more powerful Germany to defend (frugal) interests which are similar to its own. In other words, Austria opts for a position as a free-rider or fence-sitter (Schoeller & Falkner, 2022).^10^ Only if Germany deviates from its hawkish stance does Austria look to other like-minded states to form a coalition (Interviews AT1, AT4, AT6, AT7; see also Heidebrecht & Schoeller, 2022; Schoeller, 2022). In sum, contrary to our expectations, the Austrian government is hardly constrained by domestic politicisation of EU fiscal solidarity issues. If anything, we can find a form of self-constraint in which the government pro-actively makes Eurosceptic statements to mobilise voters and thus constrains itself to follow through (Interviews AT6, AT13).

### Constraint and its management in a crisis context

From the start of the COVID-19 crisis, Austria positioned itself against any joint debt initiative. Only when it became clear with the Franco-German proposal that such a fund could not be prevented altogether, did underlying substantive issues such as financing, grants versus loans and conditionality come to the fore (Interviews AT1, AT4, AT6, AT8). It was only at a later point, with the rise of the Frugal Four, that the debate became salient for the wider public and in parliament. The Franco-German proposal and the Frugal Four’s response were debated both in the parliamentary committees and in plenary, but no opinions were adopted to influence the government’s position or strategy in Brussels ex-ante. Hence, Parliament did not have any role in the emergence of the Frugal Four (Interviews AT1, AT3, AT5), but rather acted reactive by debating the government’s positions and actions. In the debate on NGEU, unsurprisingly, members of the Chancellor’s *Österreichische Volkspartei* (ÖVP) supported the positions of the Frugal Four, while two of the three opposition parties – *Sozialdemokratische Partei Österreichs* (SPÖ) and *Das Neue Österreich und Liberales Forum* (NEOS) – expressed criticism. More interestingly, the minor governing party, the Greens, and the usually very outspoken right-wing opposition party *Freiheitliche Partei Österreichs* (FPÖ), took a less pronounced stance.

In line with the Frugal Four position, members of the governing conservative party (ÖVP) demanded support based on loans rather than grants, strict conditionality, time limits, and provision of funds only in direct relation to the COVID-19 crisis and to strengthen competitiveness (Nationalrat, 2020a, pp. 3–4; 2020c, pp. 6–7). In contrast, speakers from the social-democratic SPÖ and the liberal NEOS called for more solidarity with other Member States based on grants rather than loans, and they criticised Austria’s participation in the Frugal Four as counterproductive for the country’s interests (Nationalrat, 2020a, pp. 5, 11; 2020b, pp. 4, 7; 2020d, pp. 248; 2020e, pp. 65–66, 72–74). The Greens, however, took a more reserved position. On the one hand, they were in favour of solidarity and boosting investment in the ecological transformation of Southern European economies (Nationalrat, 2020a, pp. 5). On the other hand, being the minor coalition partner, they stressed the need for conditionality (Nationalrat, 2020b, pp. 4; 2020e, pp. 76) and refrained from open criticism of the Frugal Four. Mirroring the ambiguous situation of the Greens, Members of the right-wing populist FPÖ were torn between their role in Parliament (opposition party) and their substantive preferences (close to Frugal Four), too. As opposed to the Greens, they thus implicitly endorsed the positions of the Frugal Four, as they spoke out against any kind of debt mutualisation and, in particular, against joint borrowing and financial support through grants, but criticised the result achieved in the negotiations, which in their view was poor (Nationalrat, 2020b, pp. 4–5; 2020e, pp. 62–62; also Bundesrat 2020, pp. 125). This shows that the position taken by the Austrian government mainly responded to the Eurosceptic electorate and, paradoxically, was therefore closer to the preferences of a right-wing opposition party than to those of one governing party.

As a member of the Frugal Four, Austria became a vocal opponent of fiscal solidarity. The Frugal Four coalition had already teamed up around 2017 to prevent a significant increase of the EU budget in the wake of Brexit, but they moved into the spotlight only after Germany and France had joined forces in favour of a joint EU recovery fund (Interviews AT1, AT4, AT6, AT8). Without exception, our interviewees suggested that for Chancellor Kurz, the coalition was not so much a means to reach substantial goals but rather a platform to convey the message at home that he would firmly defend Austrian’s interests against a planned debt union (Interviews AT2, AT6, AT7, AT9, AT12). Like in the Dutch case, the narrative of a looming ‘debt union’ might have served to argue that Austria’s interests were protected and to frame the final compromise in a more positive light (Politico, 2020a). But whereas the Dutch PM Rutte faced many hours of parliamentary scrutiny on the legal details of the underlying debt of NGEU and their difference with Eurobonds, Austrian Chancellor Kurz could claim victory with relative ease, arguing that the final result represented ‘a good result for Austria and the EU’ (cited in: Truchlewski et al., 2021, p. 1369).

Hence, the management of constraint takes on a markedly flexible form in the Austrian case. Given the low salience of many specific EU issues and the fact that the Austrian government is not bound by strong institutional constraints, it can pursue a very flexible EU policy, which the interviewees described as ‘situational’ (Interview AT1) or ‘anything-goes’ (Interview AT2). In fiscal governance, in particular, we do not see a direct ‘bottom-up’ process, where a trap has to be deliberately avoided or skilfully circumvented. If any, we observe a ‘top-down’ process, in which the government pro-actively places issues in the media to sharpen its profile as the defender of Austrian interests against the background of a sizeable euroskeptic voter potential, as was the case with the Frugal Four in the context of NGEU. The crisis experience did not seriously alter this. On the contrary, the described patterns seem remarkably stable, as none of our interviewees reported significant changes over time. Indeed, the dynamics described were reported to trace back even to previous SPÖ-led governments.

## Case comparison: free Austrians and shackled Dutch?

While the Dutch and Austrian cases from the outset share several similarities (e.g., Euroscepticism and opposition to fiscal solidarity), the underlying domestic political and media dynamics differ substantially. In the Netherlands, we see very assertive parliamentary scrutiny of EU affairs with clear entrapment elements, including motions voicing fundamental opposition, all in a context of high media salience. Austrian government actors are comparatively free to pick those issues they want to engage with, while many EU fiscal issues remain unnoticed by the media and public. Public opinion is only considered when decisions have been largely pre-cooked in Brussels (Interviews AT4, AT8). Regarding parliaments, it is noteworthy that the Dutch parliament punches above its weight by de facto constraining and steering government action with its motions and assertive scrutiny, even if these motions are also in part symbolic and overlapping with the government’s position. By contrast, the Austrian parliament, which has the explicit formal right to issue binding motions, largely refrains from using this instrument.

It is noticeable that in the Netherlands, an important political mechanism in adopting motions is the populist radical right’s intention (most notably the Freedom Party – PVV) to test the commitment to red lines of the centre-right coalition parties. They do so in the debate but also by proposing motions where red lines are formulated sharper than the government's official position, e.g., threatening with vetoes. For the populist radical right, there is a double-win logic as either they get support for their proposed motion, thereby further closing doors for European initiatives, or if their motion fails to find support, they can expose the centre-right coalition parties as fake and untrustworthy defenders of the national interest if pressure to compromise arises. In the case of an eventual European deal, the centre parties will have to sell it in Parliament, whereas the populist radical right can show they have been warning against this from the start. Centre-right parties, most notably the liberal VVD and Christian-Democrat CDA, respond by proposing motions themselves to set the boundaries of negotiation as a sign of responsiveness. As suspected, they perform a more difficult balancing act of setting red lines whilst also not making compromise impossible (Interviews NL2, NL3, NL4, NL5). The result is a high level of precision in the ex-ante position taking of the government. While the Austrian radical right FPÖ seeks to use this mechanism as well, if in opposition, they do so to little avail. The government parties vote down proposed motions setting tight boundaries without making a counter-proposal in the competent committee, and an effective ex post scrutiny does not take place. The reason is arguably that effective shaming as a political strategy requires an audience (Schimmelfennig et al., 2006), which, due to the low public salience of EU issues, is much smaller in Austria than in the Netherlands. The result is a considerably less constrained Austrian government.

While less constrained than the Dutch government, the Austrian government also has strong electoral incentives to prevent reputational damage through an unpopular compromise at the European level and thus lose a large share of Eurosceptic voters to the FPÖ (see Meyer, 2023). Indeed, the probability that Austrian citizens who classify themselves as right-wing will vote for the conservative ÖVP – in government during our period of study – is about as high as the probability of voting for the Eurosceptic FPÖ (Kritzinger & Wagner, 2023, p. 424). Therefore, government actors, rather than proactively setting up a positive narrative about EU integration, often choose to play up EU issues themselves by making Eurosceptic statements to gain the approval of the Eurosceptic share of voters (see Auel & Schmidt, 2022). While in the EU negotiations themselves, Austria often remains a passive fence-sitter – Austrian ministers are notorious for their absence from Council meetings^11^ and the number of Austrian abstentions is the highest of all member states (Maurer, 2023, p. 719) – former Chancellor Kurz’ self-evoked veto threat on the EU’s multiannual budget, the government’s positioning against an alleged ‘debt union’, but also the Austrian veto against the Schengen accession of Romania, can all be read in this way. While this ‘top-down politicisation’ may be an effective strategy to sharpen the government’s domestic profile, it may also lead to a loss of credibility as a coalition partner in Brussels (Interviews AT2, AT6, AT7).

Secondly, in the Netherlands media salience of European budgetary issues is in line with the strong parliamentary scrutiny, each increasing government accountability. The case of Austria is more complex. In the Press Freedom Index, Austria is in 29th place^12^, and, at the time of writing, Austria is still one of the two remaining EU countries without a freedom of information law (the other being Luxembourg). Politicians, including former Chancellor Kurz, are investigated for allegedly having used public money to buy positive news coverage. Attempts to influence the press are reported to be very frequent. For example, there have been systematically informal off-the-record meetings (*Hintergrundgespräche^13^*) with only a few invited newspapers, and politicians have reportedly contacted journalists or editorial offices to prevent certain information from being published (Interviews AT7, AT11, AT12). While this pattern has long been the case, it intensified during Kurz’s chancellorship. Under the label ‘message control’, Austrian media were divided into those who received privileged information from the government and those who were deliberately excluded from communication (Interviews AT7, AT10, AT11, AT12). This practice is sustained by the asymmetry of resources between the Chancellery, which employs more than 100 staff members for public relations alone, and the comparatively poorly equipped newspapers (Interviews AT11, AT13).^14^ Hence, the Austrian press is vulnerable to being used by the government as a means of politicising those EU issues in its interests, rather than being able to exercise effective scrutiny or constraint (see also Wodak, 2023).

All in all, Dutch government actors face significantly more constraints in their decision-making than Austrians, who sometimes spring the trap for political reasons themselves. The constant domestic political pressure pushes Dutch government actors to take a proactive role in voicing opposition and take a hard line on conditionality and budgetary rules. The Austrian government, by contrast, can afford to ‘sit on the fence’ unless it does not see its interests sufficiently protected by other member states – most notably Germany – in the case of EU fiscal governance.

## Conclusions

In this paper, we examine the role of domestic democratic constraint in European decision-making and how governments manage this element of politicisation in the face of a possible multilevel politics trap. Theoretically, we argue that the agency of governments is crucial for understanding the dynamics of the trap and potential pathways to escape it. Governments can either ‘spring the trap’ to gain bargaining power at the European level or avoid it to maintain room for finding problem-solving agreements. In both cases, they need to strategically interact with their parliament and/or the media to increase or lower the domestic audience costs of having to compromise at the European level. Whether governments spring the trap or rather avoid it ultimately depends on where they see the greater electoral advantage.

The analysis shows that constraint – while limiting options – can also be proactively managed by governments to avoid a politics trap that makes European compromise impossible because of reputational costs. In Austria, government actors keep such a possible trap under their own control. In the Netherlands, whilst the pre-NGEU period looked like a trap, the ex-ante barriers were circumvented with a parliament that ultimately, during the crisis, did not want to pull the trigger as this would lead to even higher reputational costs and a government that skilfully manoeuvred around domestic red lines. Hence, even the most likely candidates for a multilevel politics trap – the ‘Frugals’ in the case of EU fiscal governance – can manage constraints as governments can strategically interact with their parliaments and/or the media and thus increase or lower domestic audience costs. We also show how political and media culture are important elements in explaining variation in constraint, where the cases differ considerably. Dutch government agents face highly assertive scrutiny from parliamentary actors that seek to close any future opening to a compromise; in Austria, politics is more reactive in a context of smaller parliamentary influence and media salience.

Our analysis contributes to the idea that the multilevel politics trap is not a structural phenomenon where increased politicisation leads to stable political divides according to a clear causal structure, thus making the EU ungovernable in a predictable way. The pessimistic scenario of a trap is mostly agency-contingent, and even the seemingly most entrenched positions – like the Dutch – may become malleable in the face of a crisis. Importantly, Dutch and Austrian politicians have so far been mostly pragmatic, ultimately seeking cooperation rather than isolation.^15^ This is an important difference with someone like the Hungarian PM Orban, for whom electoral incentives seem to work differently. We also observe that member state coalitions in the debate on fiscal solidarity are more ad-hoc than structural. The Hanseatic League fell apart before the Covid-19 crisis. While the Frugal Four are a long-standing cooperation at the administrative level, Ministers also seek other alliances, like in the case of budgetary rules reform.

If we widen the scope, in terms of constraint, other frugal states take a middle position between our cases (except Finland, although more research would be necessary here). In Sweden, for example, parliament adopted two government statements on fiscal solidarity before the NGEU agreement, which mostly focus on principles, such as the necessity of a time-limited nature of NGEU and reform conditionality, and express criticism – but no fundamental opposition – to joint borrowing for grants (Riksdag, 2020; 2020a). Despite scepticism, this left room for the Swedish government to compromise at the European level. For Germany, Howarth and Schild (2021) find that German elites have primarily engaged in foot-dragging regarding fiscal solidarity. However, when a compromise seemed unavoidable, German governments skilfully managed to contain audience costs driven by sceptical public opinion and the rise of a challenger party. Finland, however, might represent a somewhat contrasting case that would be interesting to examine in the future. A wide majority in parliament eventually passed the NGEU agreement, but it also triggered a more fundamental legal debate, diverting political attention away from pragmatism towards constitutional principles (Leino-Sandberg, 2021). Following this debate, the 2023 incoming government introduced unequivocal language in the coalition agreement, rejecting any NGEU-like instrument, a permanent NGEU or any measure that would shape the EU into an asymmetric income transfer union (Valtioneuvosto, 2023).^16^

In conclusion, our two case studies highlight the importance of government agency when studying how domestic politicisation affects European decision-making. The polyarchic decision-making structure of the EU is full of barriers and veto points that constrain integration options. Far-reaching proposals, like a more permanent fiscal capacity, therefore, seem out of reach for the time being. Yet, our findings challenge structuralist ideas of polycrises leading to poly-cleavages, or politicisation leading to an ungovernable union, by showing political divides to be more fluid and agency-contingent. The multilevel politics trap is a pessimistic scenario, a possibility, but whether it materialises remains in the hands of governments themselves.

## Supplementary Material

Supplemental Material

## Figures and Tables

**Table:** 

Code	Interview	Date	Place
NL1	Ministry official	3 Mar 2023	Online
NL2	Ministry official	6 Mar 2023	Online
NL3	Ministry official	6 Mar 2023	Online
NL4	Parliament official	8 Mar 2023	Online
NL5	Parliament official	8 Mar 2023	Online
NL6	Politician	13 Mar 2023	Online
NL7	Journalist	13 Mar 2023	Online
AT1	Government official	27 Feb 2023	Online
AT2	Senior official	27 Feb 2023	Online
AT3	Member of Parliament (Nationalrat)	27 Feb 2023	Vienna, Austria
AT4	Senior government official	9 Mar 2023	Vienna, Austria
AT5	Member of Parliament (Nationalrat)	6 Mar 2023	In written form
AT6	Senior official	10 Mar 2023	Vienna, Austria
AT7	Journalist	13 Mar 2023	Online
AT8	Government official	20 Mar 2023	Vienna, Austria
AT9	Cabinet member (minister)	28 April 2023	Vienna, Austria
AT10	Journalist	8 May 2023	Vienna, Austria
AT11	Journalist	11 May 2023	Vienna, Austria
AT12	Journalist	17 May 2023	Vienna, Austria
AT13	Expert	22 May 2023	Vienna, Austria

## Notes

1 While we assume that government actors anticipate reputational damage in their strategic actions, it should be noted that these may rely on miscalculations. A prominent example is Dutch PM Rutte’s 2012 electoral promise that he would under no circumstances accept new funds for Greece.

2 Fiscal solidarity refers to European policy solutions that imply cross-national fiscal transfers, such as the NGEU agreement.

3 Counted from LexisNexis News analysis of Volkskrant (13), NRC (9), FD (45), AD (11), and Telegraaf (13) between October 2018 and March 2019.

4 The Finance Committee held debates with multiple mentions by numerous MPs of ‘BICC’ or ‘budgetary instrument’ on 3 April, 14 May, 11 June, 3 July, 4 September, 1 October, twice on 6 November 2019 and plenary debates on 18 June and 12 December 2019.

5 Counted between 2018 and the start of COVID-19 in April 2020 on LexisNexis Newsanalysis. Financieel Dagblad (42), Telegraaf (23), NRC Handelsblad (14), Volkskrant (10), Algemeen Dagblad (5).

6 This is corroborated by the findings of Hutter and Kriesi (2019, online appendix, Table B.2), according to which politicisation of EMU governance is immaterial in Austria (in stark contrast to the Netherlands).

7 686,431 (Krone) vs 125,733 (Kurier) (https://www.oeak.at/).

8 The right to issue opinions is flanked by hearings of the Chancellor before European Council summits and the right of parliamentarians to put questions to government members (parlamentarische Anfrage).

9
https://www.krone.at/2089782

10 This is in line with Maurer’s findings that in the period between 2009 and 2021 Austria had the highest number of abstentions in Council negotiations among all member states (2023, p. 719).

11 See e.g. https://www.derstandard.at/story/2000142272399/kaum-in-bruessel-die-konsequent-abwesende-regierung.

12
https://rsf.org/en/country/austria

13 Background talks with privileged journalists are not untypical. However, as our interviewees reported, under the Kurz government these talks took on a new dimension, as they were systematically held with only a few, supposedly government-friendly journalists and at the expense of general information events open to all journalists, thus seeking to divide the media landscape.

14 See e.g. https://www.derstandard.at/story/2000142759602/spoe-empoert-ueber-104-pr-mitarbeiter-im-kanzleramt-nehammer-sprecher; https://www.kleinezeitung.at/politik/innenpolitik/6240751/104-PRMitarbeiter_Kanzleramt-gibt-monatlich-450000-Euro-fuer. Unfortunately, it was not possible to talk to anyone in the Chancellery.

15 For both cases politics is volatile and, at the time of writing, the populist radical right is surging in polls. Historical experience shows that governments with populist parties in the Netherlands are short-lived and in Austria previous coalitions with the FPÖ did not fundamentally alter the described dynamics.

16 For Denmark, media sources (e.g. Politiken, 2020; Information, 2020) confirm the image that it was primarily PM Frederiksen who led the opposition to NGEU, with little involvement of parliament and mostly silence after the deal. We were not able to sufficiently triangulate this information with parliamentary sources or secondary literature.
