# Supplementary material for: Managing constraint: frugal opposition to European fiscal solidarity
Source: J Eur Public Policy. 2024 Mar 24;31(10):3275–98. doi: 10.1080/13501763.2024.2332697 (PMC11371260; doi:10.1080/13501763.2024.2332697)
Supplement: Supplemental Material [file RJPP_A_2332697_SM0927.docx]

**Online Appendix: Overview of Government and Parliament documents**

| **Institution** | **Code and link to document** | **Name of document** |
| --- | --- | --- |
| Dutch Parliament | [TK 2002](https://www.eerstekamer.nl/eu/behandeling/20020411/rapport_van_baalen) | van Baalen Raport ‘Op tijd is te laat’ |
| Dutch Parliament | [TK 2011](https://www.parlementairemonitor.nl/9353000/1/j9vvij5epmj1ey0/viobiu0zy5wc) | Bovenop Europa: Evaluatie van de versterkte EU-ondersteuning van de Tweede Kamer, 2007-2011 |
| Dutch Parliament | [TK 2012](https://www.tweedekamer.nl/kamerstukken/moties/detail?id=2012Z10383&did=2012D21919) | Motie van het lid Tony van Dijck over krachtig afstand nemen van het idee van Eurobonds |
| Dutch Parliament | [TK 2013](https://www.staten-generaal.nl/eu/behandeling/20131213/brief_regering_geannoteerde_agenda/document3/f=/vjfmilstb5x3.pdf) | Geannoteerde Agenda Europese Raad van 19-20 december 2013 |
| Dutch Parliament | [TK 2014](chrome-extension://efaidnbmnnnibpcajpcglclefindmkaj/https:/www.tweedekamer.nl/sites/default/files/field_uploads/Voorop%20in%20Europa_rapport_tcm181-238512_0.pdf) | Eindrapport rapporteurschap “Democratische legitimiteit”: Voorop in Europa |
| Dutch Parliament | [TK 2014a](https://www.tweedekamer.nl/kamerstukken/moties/detail?id=2014Z08650&did=2014D16970) | Motie van het lid Tony Van Dijck over met kracht afstand nemen van elke vorm van Europese belastingen en/of uitgifte van Europees schuldpapier |
| Dutch Parliament | [TK 2017](https://www.tweedekamer.nl/debat_en_vergadering/plenaire_vergaderingen/details/activiteit?id=2017A02000) | Debat over de Europese top van 22-23 juni 2017 |
| Dutch Parliament | [TK 2018](https://www.tweedekamer.nl/kamerstukken/detail?id=2018Z15055&did=2018D43448) | Verslag van een algemeen overleg, gehouden op 6 september 2018, over Eurogroep/Ecofinraad september |
| Dutch Parliament | [TK 2018a](chrome-extension://efaidnbmnnnibpcajpcglclefindmkaj/https:/www.eerstekamer.nl/eu/behandeling/20181011/brief_van_de_minister_van_2/document3/f=/vksjl4sed2zw_opgemaakt.pdf) | Verslag van de Eurogroep en Ecofinraad van 1 en 2 oktober 2018 te Luxemburg |
| Dutch Parliament | [TK 2018b](chrome-extension://efaidnbmnnnibpcajpcglclefindmkaj/https:/www.eerstekamer.nl/eu/behandeling/20181129/verslag_van_een_algemeen_overleg/document3/f=/vktvkol8r7wr.pdf) | Verslag van een algemeen overleg, gehouden op 1 november 2018, over Eurogroep/Ecofinraad november |
| Dutch Parliament | [TK 2018c](https://www.tweedekamer.nl/kamerstukken/moties/detail?id=2018D21714&did=2018D21714) | Motie van het lid Bisschop over voorbehouden en kanttekeningen bij Commissievoorstellen |
| Dutch Parliament | [TK 2018d](https://www.tweedekamer.nl/kamerstukken/commissieverslagen/detail?id=2017Z16371&did=2018D24370) | Verslag van een notaoverleg, gehouden op 26 maart 2018, over de Staat van de Europese Unie 2018 |
| Dutch Parliament | [TK 2019](https://www.tweedekamer.nl/kamerstukken/commissieverslagen/detail?id=2019Z01804&did=2019D06244) | Verslag van een algemeen overleg, gehouden op 7 februari 2019, over Eurogroep/Ecofinraad februari |
| Dutch Parliament | [TK 2019a](https://www.parlementairemonitor.nl/9353000/1/j9vvij5epmj1ey0/vkwlkxqo1cwh) | Schriftelijke toelichting Europese Commissie op SGP besluit-Italië december 2018 |
| Dutch Parliament | [TK 2019b](https://www.tweedekamer.nl/kamerstukken/moties/detail?id=2019D11521&did=2019D11521) | Motie van de leden Omtzigt en Verhoeven over openbaar maken van de memo over de begroting van Italië |
| Dutch Parliament | [TK 2019c](https://www.openkamer.org/kamervraag/2019Z04782/) | Kamervraag 2019Z04782, Het feit dat Italië het Stabiliteits-en Groeipact |
| Dutch Parliament | [TK 2019d](https://www.tweedekamer.nl/kamerstukken/kamervragen/detail?id=2019Z03784&did=2019D08072) | Schriftelijke vragen aan de minister van financiën over het opiniestuk van eurocommissaris Moscovici in de Financial Times |
| Dutch Parliament | [TK 2019e](https://www.tweedekamer.nl/debat_en_vergadering/commissievergaderingen/details?id=2019A02107) | Convocatie: Gesprek met Eurocommissaris Moscovici (economische en financiële zaken, belastingen en douane) |
| Dutch Parliament | [TK 2019f](https://www.tweedekamer.nl/kamerstukken/detail?id=2019Z11855&did=2019D24461) | Gewijzigde motie van het lid Leijten over kiezen voor een opt-out bij een begrotingsinstrument dat afwijkt van de Nederlandse voorwaarden (t.v.v. 21501-07-1608) |
| Dutch Parliament | [TK 2019g](https://www.tweedekamer.nl/kamerstukken/detail?id=2019D44285&did=2019D44285) | Verslag van een schriftelijk overleg over het BNC-fiche inzake Governanceraamwerk begrotingsinstrument voor convergentie en concurrentivermogen (BICC) |
| Dutch Parliament | [TK 2020](https://www.tweedekamer.nl/debat_en_vergadering/plenaire_vergaderingen/details/activiteit?id=2020A02629) | Debat over de Europese Top van 19 juni 2020 |
| Dutch Parliament | [TK 2020a](https://www.tweedekamer.nl/debat_en_vergadering/plenaire_vergaderingen/details/activiteit?id=2020A03073) | Debat over de Europese Top van 17 en 18 juli 2020 |
| Dutch Parliament | [TK 2020b](https://www.tweedekamer.nl/kamerstukken/moties/detail?id=2020Z06318&did=2020D13323) | Motie van het lid Omtzigt c.s. over vormen van onmiddelijke bijstand mogelijk maken |
| Dutch Parliament | [TK 2022](https://www.tweedekamer.nl/kamerstukken/detail?id=2022Z03574&did=2022D07320) | Motie van het lid Omtzigt over niet akkoord gaan met een gezamenlijke schulduitgifte voor nieuwe Europese fondsen en projecten |
| Dutch Parliament | [TK 2022a](https://www.tweedekamer.nl/kamerstukken/moties/detail?id=2022D25024&did=2022D25024) | Motie van het lid Heinen c.s. over niet instemmen met het structureel make van de Recovery and Resilience Facility |
| Dutch Parliament | [TK 2022b](https://www.tweedekamer.nl/kamerstukken/brieven_regering/detail?id=2022D12535&did=2022D12535) | Reactie op de moties van de leden Van Haga en Ephraim over niet instemmen met Eurobonds of andere vormen van schuldmutualisering (Kamerstuk 35925-167) en van het lid Omtzigt over niet akkoord gaan met een gezamenlijke schulduitgifte voor nieuwe Europese fondsen en projecten (Kamerstuk 35925-169) |
| Dutch Government | [Rijksoverheid 2022](https://www.government.nl/latest/news/2022/04/04/spain-and-the-netherlands-call-for-a-renewed-eu-fiscal-framework-fit-for-current-and-future-challenges) | Joint Eurogroup Paper: Spain and the Netherlands call for a renewed EU Fiscal Framework fit for current and future challenges |
| Dutch Parliament | [TK 2023](https://www.tweedekamer.nl/kamerstukken/moties/detail?id=2023D09932&did=2023D09932) | Gewijzigde motie van de leden Omtzigt en Stoffer over niet akkoord gaan met verdere initiatieven voor permanente gemeenschappelijke fondsen (t.v.v. 21501-20-1927) |
| Austrian Federal Chancellery | [Bundeskanzleramt 2020](https://www.bundeskanzleramt.gv.at/dam/jcr:7b9e6755-2115-440c-b2ec-cbf64a931aa8/RegProgramm-lang.pdf) | Aus Verantwortung für Österreich. Regierungsprogramm 2020-2024 |
| Austrian Federal Council | [Bundesrat 2020](https://www.parlament.gv.at/PAKT/VHG/BR/BRSITZ/BRSITZ_00907/fname_841391.pdf) | Plenarsitzung des Bundesrates. Stenographisches Protokoll. 907. Sitzung, 4 June 2020 |
| Austrian Parliament | [Nationalrat 2020a](https://www.parlament.gv.at/PAKT/VHG/XXVII/IV/IV_00003/fname_804502.pdf) | Beratungen des Hauptausschusses in Angelegenheiten der Europäischen Union. IV-03 der Beilagen zu den stenographischen Protokollen des Nationalrates XXVII. GP. Auszugsweise Darstellung, 15 June 2020 |
| Austrian Parliament | [Nationalrat 2020b](https://www.parlament.gv.at/PAKT/VHG/XXVII/IV/IV_00004/fname_810458.pdf) | Beratungen des Hauptausschusses in Angelegenheiten der Europäischen Union. IV-04 der Beilagen zu den stenographischen Protokollen des Nationalrates XXVII. GP. Auszugsweise Darstellung, 9 July 2020 |
| Austrian Parliament | [Nationalrat 2020c](https://www.parlament.gv.at/PAKT/VHG/XXVII/V/V_00003/fname_812681.pdf) | Beratungen des Ständigen Unterausschusses in Angelegenheiten der Europäischen Union. V-3 der Beilagen zu den stenographischen Protokollen des Nationalrates XXVII. GP. Auszugsweise Darstellung, 1 July 2020 |
| Austrian Parliament | [Nationalrat 2020d](https://www.parlament.gv.at/PAKT/VHG/XXVII/NRSITZ/NRSITZ_00032/fname_839887.pdf) | Plenarsitzung des Nationalrates. Stenographisches Protokoll. 32. Sitzung, 26-29 May 2020 |
| Austrian Parliament | [Nationalrat 2020e](https://www.parlament.gv.at/PAKT/VHG/XXVII/NRSITZ/NRSITZ_00036/fname_844432.pdf) | Plenarsitzung des Nationalrates. Stenographisches Protokoll. 36. Sitzung, 17 June 2020 |
| Swedish Parliament | [Riksdag 2020](https://www.riksdagen.se/sv/dokument-och-lagar/dokument/utskottens-protokoll/protokoll-utskottssammantrade-20192057_h7a1fiu57p/) | Protokoll utskottssammanträde 2019/20:57 |
| Swedish Parliament | [Riksdag 2020a](https://www.riksdagen.se/sv/dokument-och-lagar/dokument/utskottens-protokoll/protokoll-utskottssammantrade-20192061_h7a1fiu61p/) | Protokoll utskottssammanträde 2019/20:61 |
| Finnish Government | [Valtioneuvosto 2023](https://kuvapankki.valtioneuvosto.fi/l/88PZvqzQ6hVG) | Excerpt outcome oft he negotiations on the government programme 16 June 2023 |
